# Structural Refinement and Enhanced Interfacial Electrochemical Properties of Ultrasonic-Assisted Molasses-Derived LaFeO_3_ Nanoperovskites

**DOI:** 10.3390/molecules31152707

**Published:** 2026-08-04

**Authors:** José G. Alfonso-Gonzalez, Valentina Toro-Corrales, Luz E. Renteria-Moreno, Jimmy A. Morales-Morales

**Affiliations:** Grupo de Investigación en Química y Biotecnología (QUIBIO), Facultad de Ciencias Básicas, Universidad Santiago de Cali, Cali 760035, Colombia

**Keywords:** LaFeO_3_ nanoperovskites, sugarcane molasses, electrochemical interfaces, ultrasonic-assisted synthesis

## Abstract

LaFeO_3_ nanoperovskites were synthesized through a sugarcane-molasses-assisted combustion route using mechanically stirred (MLP) and ultrasonic-assisted (ULP) activation strategies to investigate the influence of synthesis conditions on structural and interfacial electrochemical properties. X-ray diffraction and Rietveld refinement confirmed the formation of orthorhombic LaFeO_3_, while ultrasonic-assisted synthesis promoted improved phase homogeneity and reduced crystallite size compared with mechanically stirred combustion. Transmission electron microscopy revealed lower agglomeration and improved particle dispersion for ULP materials, whereas thermal and vibrational analyses confirmed the formation of thermally stable LaFeO_3_ nanoperovskites containing residual biomass-derived species associated with the combustion process. Electrochemical characterization at screen-printed carbon electrodes demonstrated that ultrasonically synthesized LaFeO_3_ significantly enhanced interfacial charge-transfer behavior, yielding lower charge-transfer resistance (435 Ω), increased electroactive surface area (0.149 cm^2^), and improved heterogeneous electron-transfer kinetics relative to MLP and bare electrodes. The LaFeO_3_-modified interfaces additionally exhibited distinct electrochemical oxidation behavior toward 2-aminothiazole (2AT) and 2-aminooxazole (2AO) under acidic conditions. Scan-rate analyses revealed predominantly diffusion-controlled irreversible oxidation processes, while pH-dependent studies indicated proton-coupled electron-transfer behavior during electrooxidation. The combined structural and electrochemical results establish clear process–structure–property relationships linking ultrasonic-assisted green synthesis, nanostructural organization, and interfacial electrochemical performance in LaFeO_3_ nanoperovskites.

## 1. Introduction

Perovskite-type oxides with the general formula ABO_3_ have attracted considerable attention owing to their remarkable structural flexibility, physicochemical stability, and tunable electronic properties, which make them highly attractive for electrochemical and catalytic applications [1,2,3]. In particular, lanthanum-based perovskites have demonstrated promising charge-transfer capabilities associated with the redox activity of transition-metal cations, oxygen ion mobility, and adaptable crystal structures [4,5]. Among them, LaFeO_3_ has emerged as an important electroactive material due to its semiconducting behavior, chemical stability, and favorable interfacial electron-transfer properties [2,6,7]. These characteristics have promoted its application in electrocatalysis, sensing platforms, energy conversion, and electrochemical interfaces [4,7]. Furthermore, the electrochemical performance of LaFeO_3_ strongly depends on its crystallographic features, particle dimensions, surface characteristics, and synthesis methodology, making the development of structurally controlled nanoperovskites highly relevant for advanced electrochemical applications [7,8].

Different synthetic strategies have been explored to tailor the structural and electrochemical properties of LaFeO_3_ nanomaterials, including sol–gel [9,10,11,12,13,14,15], hydrothermal [10,16,17], combustion [18,19,20,21,22], co-precipitation [23], and microwave-assisted routes [24]. Among these methods, combustion synthesis has emerged as an attractive approach because of its simplicity, low processing time, energy efficiency, and ability to produce highly crystalline oxide materials. In recent years, increasing attention has been directed toward environmentally friendly synthesis methodologies employing renewable organic precursors and bio-derived fuels [15,17,23,25,26,27]. In this context, sugarcane molasses represents a low-cost and sustainable carbon-rich precursor containing carbohydrates, minerals, and organic compounds that can promote homogeneous combustion processes and influence particle growth during oxide formation [19,20,21,22,28]. Additionally, ultrasonic-assisted synthesis has been recognized as an effective strategy for improving precursor dispersion, enhancing mass transfer, and modifying nucleation and growth mechanisms through acoustic cavitation effects [29,30,31,32,33]. These phenomena can significantly influence crystallinity, particle size distribution, surface characteristics, and interfacial electrochemical behavior of nanoperovskite materials [34].

Although LaFeO_3_-based nanomaterials have been extensively investigated for electrochemical and catalytic applications [32,35,36], several challenges still remain regarding the establishment of clear relationships between synthesis methodology, structural characteristics, and interfacial electrochemical behavior [37]. In particular, studies involving environmentally friendly combustion approaches assisted by ultrasound are still limited, despite the recognized advantages of sonochemical methods for promoting homogeneous nucleation, particle size reduction, and enhanced mass transfer during nanomaterial synthesis [38]. Furthermore, many previously reported studies have primarily focused on phase identification and qualitative electrochemical observations, while comprehensive structural refinement and detailed interfacial electrochemical analyses remain comparatively scarce [39]. Quantitative crystallographic evaluation through Rietveld refinement, combined with electrochemical kinetic analysis and mechanistic interpretation, can provide valuable insights into how synthesis-induced structural variations influence electron-transfer processes in nanoperovskite systems [38]. Therefore, developing sustainable synthetic approaches capable of modulating the structural and electrochemical properties of LaFeO_3_ nanomaterials remains highly relevant for advanced electrochemical applications.

In this work, LaFeO_3_ nanoperovskites were synthesized through an ultrasonic-assisted green combustion approach using sugarcane molasses as a renewable organic precursor. The influence of the ultrasonic-assisted synthesis route on the structural and interfacial electrochemical properties of the obtained materials was systematically investigated. Crystalline features and microstructural characteristics were evaluated by X-ray diffraction combined with Rietveld refinement analysis. The electrochemical interfacial behavior of the modified electrodes was studied by cyclic voltammetry and electrochemical impedance spectroscopy using ferri/ferrocyanide redox probes, while the electrooxidation of 2-aminothiazole and 2-aminooxazole was explored through electrochemical kinetic analysis. The obtained results provide valuable information regarding the relationship between sustainable synthesis strategies, structural characteristics, and electrochemical interfacial performance in LaFeO_3_ nanoperovskite systems

## 2. Results and Discussion

Two LaFeO_3_ samples were synthesized through combustion-assisted routes using sugarcane molasses as a biomass-derived fuel source. The first material was obtained by conventional thermal combustion (MLP), while the second was prepared under ultrasonic-assisted combustion conditions (ULP). The synthesis parameters and experimental conditions employed for both materials are summarized in Table 1.

### 2.1. Structural Refinement and Crystalline Properties

**Figure 1:**
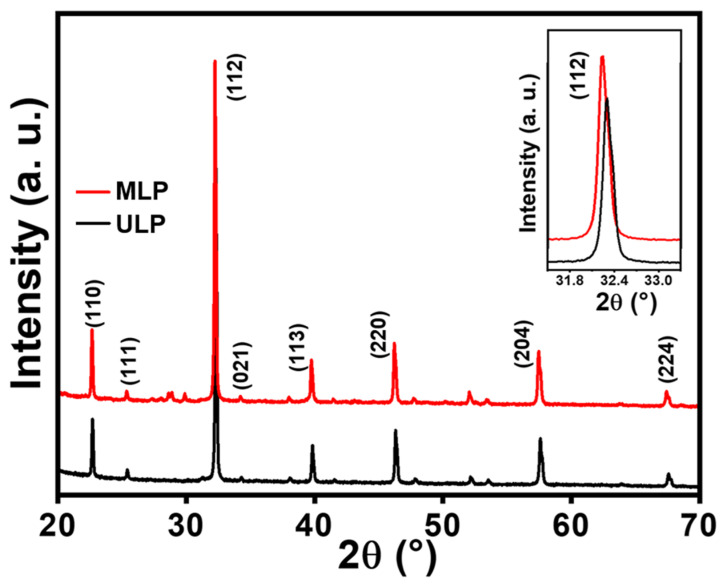
Experimental X-ray diffraction (XRD) patterns comparison of LaFeO_3_ nanoparticles synthesized through ultrasonic-assisted combustion (ULP) and mechanically stirred combustion (MLP). Inset/zoom peak broadening highlights subtle differences in peak width and relative intensity between both samples.

Figure 1 presents the X-ray diffraction patterns of the LaFeO_3_ materials synthesized through mechanically stirred combustion (MLP) and ultrasonic-assisted combustion (ULP) routes

The diffraction peaks observed for both samples can be indexed to the orthorhombic perovskite structure of LaFeO_3_ with Pnma space group symmetry, in agreement with the corresponding crystallographic reference pattern (ICDD PDF No. 37-1493) [25,29,40]. The ULP sample exhibited well-defined diffraction peaks associated exclusively with the orthorhombic LaFeO_3_ phase [41,42,43], whereas the MLP material presented minor additional reflections attributable to low concentrations of La_2_O_3_ and La(OH)_3_ secondary phases. These results suggest that ultrasonic irradiation promoted improved precursor homogenization and enhanced reaction uniformity during the combustion process, favoring the formation of highly crystalline LaFeO_3_ nanoperovskites. Slight differences in peak broadening and relative intensities were also observed between MLP and ULP samples, indicating modifications in crystallite dimensions and microstructural characteristics induced by the ultrasonic-assisted combustion process [29,30].

**Figure 2:**
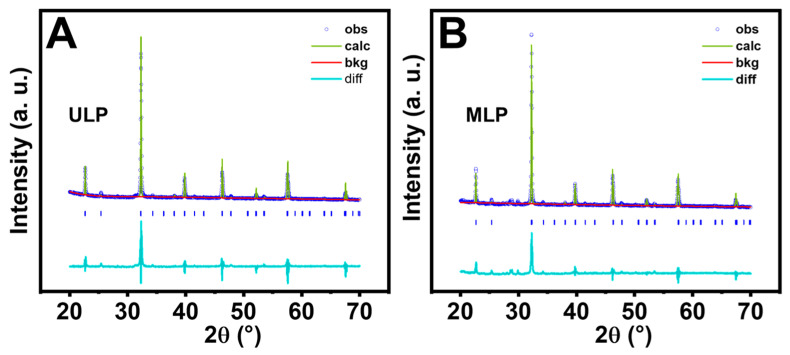
Rietveld refinement profiles of orthorhombic LaFeO_3_ synthesized by (**A**) ultrasonic-assisted combustion (ULP) and (**B**) mechanically stirred combustion (MLP). Experimental data are shown as blue open circles, calculated profiles as green lines, background contributions as red lines, and difference curves as cyan traces. Vertical tick marks indicate the calculated Bragg reflection positions of orthorhombic LaFeO_3_.

To obtain a more detailed structural evaluation, the diffraction data were analyzed by Rietveld refinement using the orthorhombic Pnma structural model of LaFeO_3_ [42,44]. Rietveld refinement confirmed the formation of orthorhombic LaFeO_3_ in both samples. As shown in Figure 2A,B, the refined profiles exhibited satisfactory agreement between observed and calculated diffraction patterns, yielding refinement indicators of Rw = 15.34% and χ^2^ = 23.81 for ULP, and Rw = 12.77% and χ^2^ = 15.05 for MLP. These results support the reliability of the orthorhombic LaFeO_3_ structural model for both synthesis routes [45].

The refinement converged successfully for both MLP and ULP samples, confirming the predominance of the orthorhombic LaFeO_3_ phase [43,44,46,47]. The refined crystallographic parameters are summarized in Table 2. Slight variations in lattice parameters and unit-cell volume were observed between the ULP and MLP samples, suggesting subtle synthesis-induced structural modifications associated with the distinct combustion environments generated under ultrasonic irradiation. Interestingly, the refinement converged successfully for both samples. Although MLP exhibited slightly lower refinement residuals, ULP showed smaller crystallite size and improved phase purity according to the diffraction profiles. In addition, ULP displayed smaller average crystallite size, consistent with the ability of ultrasonic irradiation to promote rapid nucleation while limiting excessive crystal growth during combustion synthesis. Similar structural variations have previously been reported for sonochemically assisted oxide systems, where ultrasonic irradiation can affect nucleation kinetics and crystalline development [48,49].

The average crystallite sizes estimated from the diffraction profiles were 70.62 nm for ULP and 86.01 nm for MLP, confirming that ultrasonic irradiation effectively suppressed excessive crystal growth during combustion synthesis. The corresponding diffraction peak positions, FWHM values, and crystallite-size estimations obtained from representative reflections are summarized in Table 3.

The crystallite sizes obtained in the present work were further compared with representative LaFeO_3_ synthesis routes reported in the literature (Table 4).

Although several wet-chemical and hydrothermal methods produce smaller LaFeO_3_ crystallites than the present combustion route, reduced crystallite size alone does not necessarily imply superior electrochemical performance. Interfacial behavior also depends on phase purity, particle agglomeration, interparticle connectivity, electroactive area, and charge-transfer resistance. Therefore, direct performance comparisons among the synthesis routes listed in Table 4 should be made cautiously because the reported materials were obtained and evaluated under different experimental conditions. The relevance of the present ultrasonic-assisted biomass combustion route lies in combining a renewable molasses-derived fuel, phase-pure orthorhombic LaFeO_3_, reduced crystallite size relative to the mechanically stirred combustion route, and quantitative structural and interfacial electrochemical characterization.

The acoustic cavitation generated under ultrasonic conditions can promote localized high-energy microenvironments, enhancing nucleation rates while limiting excessive crystal growth and agglomeration [29,49]. Such effects have been widely associated with the formation of nanostructured oxide materials possessing improved surface accessibility and interfacial characteristics [29,59].

Overall, the structural and microstructural analyses demonstrate that the incorporation of ultrasonic irradiation during combustion synthesis significantly influences the crystallization behavior and nanostructural characteristics of LaFeO_3_ materials. The refined crystallographic parameters and reduced crystallite dimensions observed for the ultrasonically synthesized sample suggest the formation of more accessible nanostructured interfaces, which may directly affect the interfacial charge-transfer properties and electrochemical response of the obtained nanoperovskites [60].

### 2.2. Morphological and Surface Characteristics

**Figure 3:**
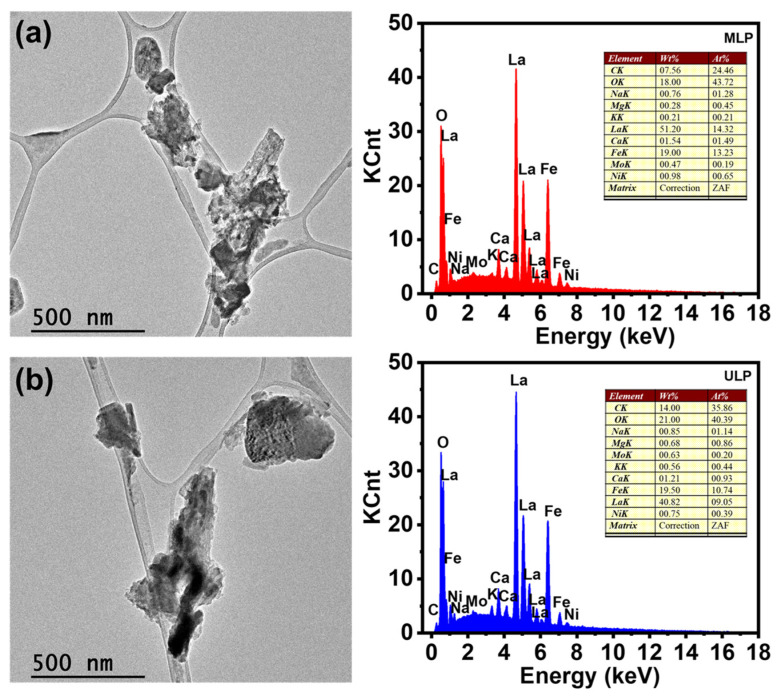
TEM micrographs and corresponding EDS spectra, of LaFeO_3_ nanoparticles synthesized through (**a**) ultrasonic-assisted combustion (ULP) and (**b**) mechanically stirred combustion (MLP). The inset tables summarize the elemental compositions obtained from EDS analysis.

The morphological and compositional characteristics of the synthesized LaFeO_3_ materials were investigated by transmission electron microscopy (TEM) and energy-dispersive X-ray spectroscopy (EDS) in order to evaluate the influence of the synthesis route on particle organization and surface features. Representative TEM micrographs and EDS spectra of the MLP and ULP samples are presented in Figure 3. Both materials exhibited irregularly shaped agglomerated nanoparticles, which is commonly observed in combustion-derived oxide systems due to the rapid gas evolution and localized thermal gradients generated during the combustion process [61,62]. Nevertheless, noticeable differences in particle distribution and agglomeration behavior were observed between the two synthesis routes. The MLP sample displayed larger and more compact agglomerates, whereas the ULP material exhibited less compact agglomerated domains and greater spatial separation among particle assemblies. Both samples formed irregular agglomerated assemblies within the approximate size range of 50–300 nm, suggesting that the observed structures consist of multiple interconnected nanocrystalline domains. The 50–300 nm dimensions observed by TEM correspond to agglomerated assemblies containing multiple nanocrystalline domains and should not be interpreted as primary crystallite sizes. Exploratory DLS measurements yielded hydrodynamic diameters of 1.076 µm for ULP and 1.389 µm for MLP, with polydispersity indices of 25.9% and 26.3%, respectively. These micrometre-scale values indicate that the calcined nanocrystalline domains form larger agglomerated assemblies in aqueous suspension. Because the measurements were exploratory and the dispersions exhibited low transmittance and visible re-agglomeration, the hydrodynamic diameters were not interpreted as primary particle sizes or directly compared with the crystallite dimensions obtained by XRD/Rietveld refinement. TEM analysis also revealed heterogeneous particle morphologies with partially amorphous surface regions, a feature commonly observed in combustion-derived perovskite oxide systems. These observations are consistent with the crystallite dimensions estimated from the XRD and Rietveld analyses discussed previously.

The observed morphological differences suggest that ultrasonic irradiation significantly influenced the nucleation and particle growth processes during combustion synthesis. Acoustic cavitation generated under ultrasonic conditions can produce localized high-temperature and high-pressure microenvironments capable of accelerating nucleation while limiting excessive particle growth and agglomeration [63]. Additionally, the formation of microbubbles and shock waves during sonochemical treatment may contribute to the lower compaction of the agglomerated domains observed for ULP [64]. These morphological observations are qualitative; their possible relationship with interfacial behavior is evaluated in Section 2.4 using the measured electroactive area and charge-transfer resistance.

EDS analysis confirmed the presence of La, Fe, and O as the principal elemental constituents of both synthesized materials, consistent with the formation of LaFeO_3_ nanoperovskites. In addition to the major elements, trace amounts of Na, Mg, K, and Ca were detected in both samples, which are attributed to residual inorganic constituents naturally present in sugarcane molasses used during the combustion process. Carbon was also detected in both materials, suggesting the presence of biomass-derived carbonaceous surface species associated with the green synthesis route. Notably, the ultrasonically synthesized ULP sample exhibited a higher carbon contribution than the MLP material, indicating differences in the retention of combustion-derived surface species. Similar residual carbon contributions have previously been reported in biomass-assisted syntheses of perovskite oxides and related nanostructured materials [65,66,67]. ICP-OES analysis further confirmed the approximate stoichiometric relationship between La and Fe for both synthesized materials, supporting the successful formation of LaFeO_3_ nanoperovskites. Minor compositional variations are commonly observed in combustion-derived oxide systems due to the complex thermal environments generated during synthesis.

Overall, the TEM and EDS analyses demonstrate that ultrasonic-assisted combustion promotes the formation of more dispersed nanostructured LaFeO_3_ materials with reduced agglomeration and modified surface characteristics. The higher carbon signal detected for ULP indicates a greater contribution of residual biomass-derived carbon-containing surface species. However, EDS does not establish their bonding state, spatial distribution, or electrical role; therefore, these species should not be interpreted as a continuous conductive carbon coating without additional surface-sensitive evidence such as XPS.

The improved electrochemical behavior observed for ULP is discussed in relation to its smaller crystallite size, less compact agglomerated domains, lower charge-transfer resistance, and larger electroactive area, as quantified in Section 2.4. Nevertheless, a potential contribution of biomass-derived surface species to interfacial charge-transfer processes cannot be excluded and warrants further investigation using surface-sensitive characterization techniques. Consequently, the influence of these structural and surface characteristics on the electrochemical behavior of the synthesized materials was further investigated by electrochemical techniques.

### 2.3. Thermal and Vibrational Features

**Figure 4:**
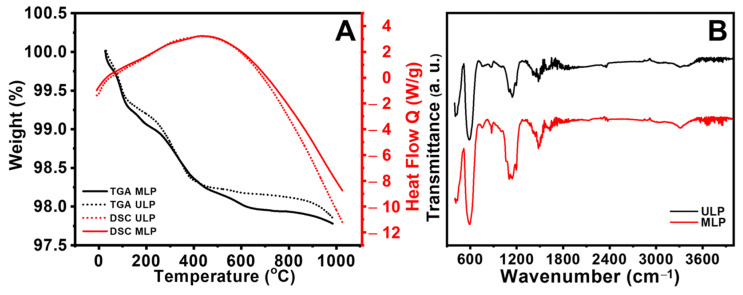
(**A**) Thermogravimetric/differential scanning calorimetry (TGA/DSC) curves and (**B**) FTIR spectra of LaFeO_3_ nanoparticles synthesized through ultrasonic-assisted combustion (ULP) and mechanically stirred combustion (MLP) respectively.

The thermal behavior of the synthesized LaFeO_3_ materials was investigated by thermogravimetric analysis (TGA) and differential scanning calorimetry (DSC), and the corresponding curves are presented in Figure 4A.

Both MLP and ULP samples exhibited an initial mass-loss region below approximately 400 °C, corresponding to an overall weight loss of approximately 1.7%, which can be attributed to the removal of physically adsorbed water and low-molecular-weight organic species originating from the sugarcane molasses precursor. Because both materials had previously been calcined at 900 °C and XRD confirmed crystalline LaFeO_3_ before thermal analysis, the exothermic DSC contribution below 400 °C is not assigned to oxide formation, but to the oxidation and decomposition of residual biomass-derived organic species. The slight divergence between the MLP and ULP curves above approximately 500 °C, associated with a mass variation below 0.3% and a weak higher-temperature thermal contribution, may reflect differences in the amount and nature of residual carbon-containing, hydroxyl-related, and other oxygen-containing surface species retained after synthesis. This minor variation is not interpreted as evidence of a bulk phase transformation. Above 600 °C, no significant additional thermal events were detected, indicating the successful formation of thermally stable LaFeO_3_ perovskite phases under the applied calcination conditions, in agreement with previous reports on calcined LaFeO_3_ systems [68].

Fourier-transform infrared (FTIR) spectra of the synthesized materials are presented in Figure 4B. Characteristic absorption bands located near 593 and 419 cm^−1^ were assigned to Fe–O stretching and O–Fe–O bending vibrations associated with FeO_6_ octahedra, confirming the formation of the LaFeO_3_ perovskite structure [68]. Broad absorption bands centered around 3420 and 1630 cm^−1^ were attributed to O–H stretching and bending vibrations of adsorbed water molecules and surface hydroxyl groups. Weak absorption features were observed near 756, 868, 992, and 1093 cm^−1^. Because the 700–1100 cm^−1^ region may contain overlapping contributions from adsorbed carbonate species and C–O vibrations of residual biomass-derived compounds, these bands cannot be assigned unambiguously to a single surface species based on FTIR alone. They are therefore discussed only as evidence of residual carbon-containing surface contributions, whose precise chemical identity remains unresolved.

Interestingly, the vibrational profiles of the ultrasonically synthesized ULP material exhibited slightly more intense absorption features associated with residual surface species compared with the MLP sample, suggesting a comparatively higher contribution of biomass-derived surface species. Such behavior is consistent with the TEM and EDS observations, indicating a greater surface contribution of biomass-derived carbonaceous species in the ultrasonically synthesized material. In contrast, the MLP material exhibited comparatively weaker intermediate absorption bands, suggesting a lower contribution of residual surface species after calcination. Residual carbon-containing surface species and their influence on interfacial properties have been previously discussed for LaFeO_3_-based oxide systems and related carbon-containing nanocomposites [65,66].

Overall, the thermal and vibrational analyses confirm the successful formation of thermally stable LaFeO_3_ nanoperovskites while revealing the presence of residual surface species associated with the biomass-assisted combustion process.

### 2.4. Interfacial Electrochemical Properties

**Figure 5:**
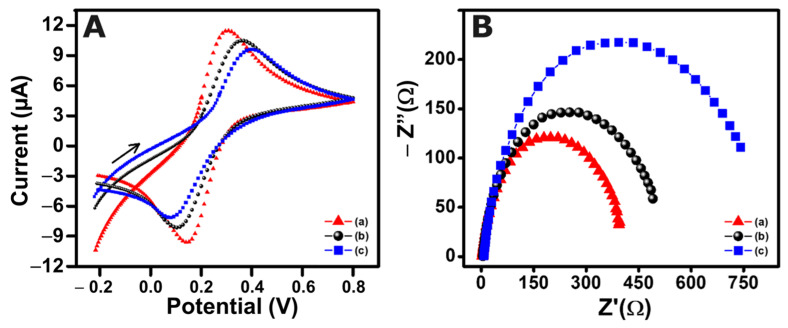
(**A**) Cyclic voltammograms and (**B**) Nyquist plots obtained for LaFeO_3_ nanoparticles synthesized through ultrasonic-assisted combustion (ULP) and mechanically stirred combustion (MLP) respectively (a) ULP/SPCE, (b) MLP/SPCE, and (c) bare SPCE in 0.1 M KCl containing 5.0 × 10^−4^ mol L^−1^ [Fe(CN)_6_]^3−^/^4−^. The inset in (**A**) shows enhanced redox current peaks for the ULP/SPCE, while B highlights reduced semicircle diameters corresponding to lower charge-transfer resistance. The arrow indicates the direction of the potential sweep.

The interfacial electrochemical behavior of the fabricated electrodes was investigated by cyclic voltammetry (CV) and electrochemical impedance spectroscopy (EIS) using the [Fe(CN)_6_]^3−^/^4−^ redox probe in 0.1 M KCl, see Figure 5.

Figure 5A presents the cyclic voltammograms obtained for bare SPCE, MLP/SPCE, and ULP/SPCE at a scan rate of 0.1 V s^−1^. Both modified electrodes exhibited enhanced anodic and cathodic peak currents compared with the bare SPCE, indicating improved electron-transfer properties after modification with LaFeO_3_ nanoperovskites. Among the evaluated systems, the ultrasonically synthesized ULP/SPCE electrode displayed the highest redox current response, suggesting more favorable interfacial charge-transfer behavior. The interfacial differences among the evaluated electrodes were quantified by EIS and scan-rate analysis through the charge-transfer resistance and electroactive area, respectively.

Electrochemical impedance spectroscopy was employed to further investigate the interfacial kinetics of the modified electrodes under identical experimental conditions (Figure 5B). The Nyquist plots exhibited characteristic semicircular regions at high frequencies associated with charge-transfer resistance (*R_ct_*), followed by diffusion-controlled behavior at lower frequencies. The bare SPCE electrode presented the largest semicircle diameter, corresponding to an *R_ct_* value of approximately 880 Ω, indicating comparatively slower electron-transfer kinetics at the electrode/electrolyte interface. Modification with LaFeO_3_ nanoparticles significantly reduced the interfacial resistance values, particularly for the ultrasonically synthesized material. The ULP/SPCE electrode exhibited the lowest *R_ct_* value (435 Ω), whereas the MLP/SPCE system presented an intermediate resistance value of approximately 585 Ω. The enhanced interfacial response is discussed primarily in relation to the lower *R_ct_*, larger electroactive area, reduced crystallite dimensions, and lower agglomeration of ULP. A contribution from residual biomass-derived surface species cannot be excluded, but it cannot be quantified from the present data. These results confirm that ultrasonic-assisted combustion promoted the formation of nanostructured interfaces with improved charge-transfer characteristics and enhanced electrolyte accessibility. The improved interfacial charge-transfer properties observed for the ultrasonically synthesized material may also be associated with subtle synthesis-induced structural distortions and modified local electronic environments commonly reported in combustion-derived perovskite oxides.

The electrochemical response was further evaluated as a function of scan rate in order to investigate the charge-transfer mechanism and estimate the electroactive surface area of the electrodes. The anodic peak current exhibited a linear dependence on the square root of the scan rate (*v*^1^ᐟ^2^), indicating that the redox process is predominantly diffusion-controlled. The electroactive surface areas were estimated using the Randles–Ševčík equation (Equation (1)) [69,70,71].

(1) $$I_{p}=2.69\;\times \;10^{5}AC\sqrt{n^{3}Dv}$$

The calculated electroactive areas were 0.084 cm^2^ for bare SPCE, 0.121 cm^2^ for MLP/SPCE, and 0.149 cm^2^ for ULP/SPCE, demonstrating that ULP/SPCE exhibited the largest electroactive area among the evaluated electrodes.

The heterogeneous electron-transfer rate constants (*k*^0^) were additionally estimated from the *R_ct_* values using Equation (2).

(2) $${Kk}^{0}=\frac{RT}{F^{2}R_{ct}AC}$$

The calculated *k*^0^ values were 1.62 × 10^−6^ cm s^−1^ for MLP/SPCE, 4.62 × 10^−6^ cm s^−1^ for the bare SPCE, and 9.8 × 10^−6^ cm s^−1^ for ULP/SPCE. The substantially higher kinetic constant obtained for the ULP/SPCE electrode further demonstrates that ultrasonic-assisted synthesis significantly improved the interfacial electron-transfer kinetics of the LaFeO_3_-modified electrodes. The higher *k*^0^ value of ULP/SPCE is consistent with its lower Rct and larger electroactive area.

Overall, the electrochemical characterization demonstrates that the incorporation of ultrasonically synthesized LaFeO_3_ nanoperovskites significantly improves the interfacial electrochemical properties of SPCE-based electrodes. The combined CV, EIS, and scan-rate analyses indicate that the enhanced electrochemical response of the ULP/SPCE system is closely associated with its reduced crystallite dimensions, improved particle dispersion, and increased electroactive surface area, confirming the effectiveness of ultrasonic-assisted biomass combustion for the preparation of electrochemically active nanoperovskite interfaces.

### 2.5. Electrochemical Oxidation Behavior of Heterocyclic Compounds

**Figure 6:**
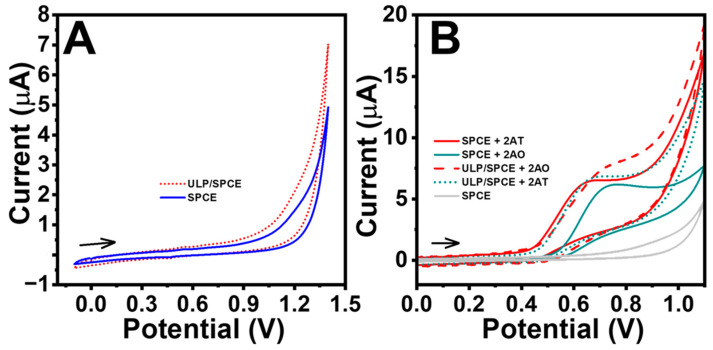
Cyclic voltammetric profiles recorded during forward and reverse potential scans: (**A**) bare SPCE and ULP/SPCE in 0.1 M phosphate buffer (pH 2.5) at a scan rate of 25 mV s^−1^; and (**B**) bare SPCE and ULP/SPCE in 0.1 M phosphate buffer (pH 6.0) containing 5.0 × 10^−4^ mol L^−1^ 2-aminothiazole (2AT) or 2-aminooxazole (2AO) at a scan rate of 0.1 V s^−1^. No corresponding cathodic peak was observed for either analyte on the reverse sweep.

The electrochemical oxidation behavior of 2-aminothiazole (2AT) and 2-aminooxazole (2AO) was investigated at bare SPCE and ultrasonically synthesized LaFeO_3_-modified electrodes (ULP/SPCE) under optimized experimental conditions. Figure 6 presents the cyclic voltammetric responses obtained in 0.1 M phosphate buffer solution (pH 6.0) containing 5.0 × 10^−4^ mol L^−1^ of each heterocyclic compound. Compared with bare SPCE, the ULP/SPCE electrode promoted distinct oxidation responses for both heterocyclic compounds, producing lower oxidation overpotentials and molecule-dependent changes in current response [72]. These observations are consistent with the enhanced electroactive surface area and improved interfacial charge-transfer properties previously identified by EIS analyses.

Distinct electrochemical responses were observed between 2AT and 2AO at the ULP/SPCE electrode. The complete forward and reverse potential scans were recorded; however, no corresponding cathodic peaks were detected on the reverse sweep, indicating irreversible oxidation under the evaluated conditions. The oxidation of 2AO exhibited a more pronounced increase in anodic current and a larger negative shift in oxidation potential relative to the bare SPCE, whereas 2AT displayed comparatively broader oxidation responses. The anodic peak current and potential values extracted from the cyclic voltammetric profiles are summarized in Table 5.

The oxidation peak potential of 2AO decreased from 0.755 ± 0.026 V at bare SPCE to 0.686 ± 0.031 V at ULP/SPCE, accompanied by an increase in anodic current from 3.16 ± 0.29 µA to 3.75 ± 0.34 µA. In contrast, 2AT exhibited a positive shift in oxidation potential from 0.663 ± 0.034 V to 0.725 ± 0.021 V after modification with ULP, although with broader anodic features. ULP/SPCE promoted differentiated oxidation behavior, enhancing the response toward 2AO while modifying the oxidation pathway of 2AT. Such differentiated electrochemical behavior suggests molecule-dependent interfacial interactions between the heterocyclic structures and the LaFeO_3_-modified surface [74].

The inter-electrode relative standard deviations ranged from 8.0% to 12.6% for the anodic peak current and from 2.9% to 5.1% for the peak potential. These results indicate comparatively reproducible peak potentials and moderate electrode-to-electrode variability in current response for the disposable drop-cast SPCE interfaces. The highest current variability was observed for 2AT at ULP/SPCE, consistent with its broader oxidation response and greater susceptibility to interfacial passivation.

Progressive attenuation of the oxidation peak during consecutive voltammetric cycling suggests the formation of adsorbed oxidation products and secondary chemical transformations at the electrode interface. Such behavior is consistent with previous reports describing electrooxidative coupling and oligomerization phenomena in amino-substituted thiazole systems. Notably, signal decay was substantially faster for 2AT than for 2AO, indicating molecule-dependent interfacial reaction pathways and behavior consistent with stronger surface passivation by 2AT oxidation products. Therefore, regeneration and reuse of the modified SPCEs cannot be assumed under the experimental conditions evaluated.

**Figure 7:**
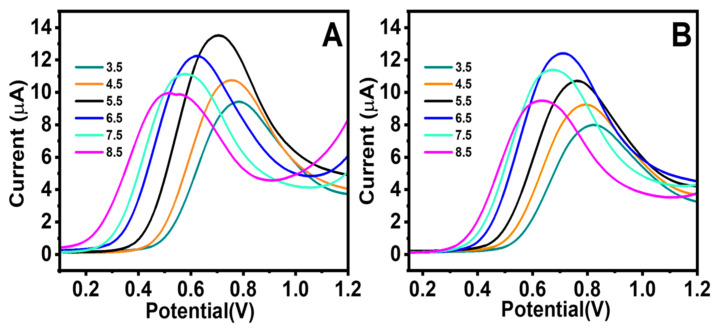
Differential pulse voltammograms (DPV) recorded at ULP/SPCE in phosphate buffer of varying pH values (3.5–8.5) containing 5.0 × 10^−4^ mol L^−1^ of (**A**) 2-aminothiazole (2AT) and (**B**) 2-aminooxazole (2AO).

Figure 7 shows the differential pulse voltammetry (DPV) responses of ULP/SPCE for 5.0 × 10^−4^ mol L^−1^ 2AT and 2AO in phosphate buffer between pH 3.5 and 8.5. As the medium became less acidic, the peak currents progressively decreased, and the oxidation potentials shifted toward less positive values, indicating that proton-coupled electron transfer (PCET) plays an important role during anodic oxidation of both heterocyclic compounds.

Linear relationships between oxidation potential and pH were obtained for both compounds, with slopes close to 56–59 mV pH^−1^, suggesting the participation of comparable numbers of electrons and protons in the electrochemical oxidation mechanism [72,75]. The corresponding linear relationships were Ep = −0.055 pH + 0.9914 (R^2^ = 0.9959) for 2AT and Ep = −0.039 pH + 1.0356 (R^2^ = 0.9963) for 2AO. Although higher anodic currents were observed under strongly acidic conditions, extreme pH values were avoided because excessive proton concentration and highly alkaline media may compromise interfacial stability and electrochemical reproducibility. Therefore, pH 6.0 was selected for subsequent electrochemical analyses because it provided a suitable compromise between signal intensity, interfacial stability, and proximity to the analyte pKa values (~5.3–5.5), where protonation/deprotonation equilibria are expected to influence the oxidation process.

**Figure 8:**
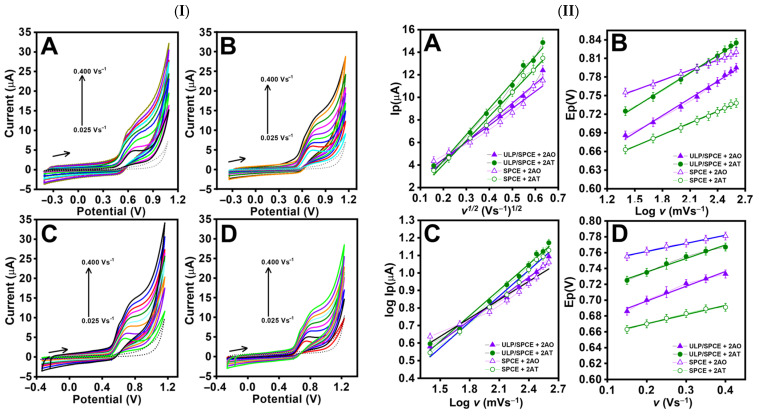
(**I**) Cyclic voltammetric profiles recorded during forward and reverse potential scans for 5.0 × 10^−4^ mol L^−1^: (**IA**) ULP/SPCE + 2AO, (**IB**) SPCE + 2AO, (**IC**) ULP/SPCE + 2AT, and (**ID**) SPCE + 2AT in 0.1 M PBS (pH 6.0) at scan rates ranging from 0.025 to 0.40 V s^−1^. No corresponding cathodic peaks were observed on the reverse sweeps. (**II**) Linear plots of (**IIA**) Ipa vs. *v*^1^ᐟ^2^, (**IIB**) Epa vs. log *v*, (**IIC**) log Ipa vs. log *v*, and (**IID**) Epa vs. *v* for both electrodes.

The influence of scan rate on the electrochemical response was further evaluated in order to investigate oxidation kinetics at the modified electrode interface. Figure 8I,II presents the cyclic voltammograms recorded between 0.025 and 0.40 V s^−1^ for both analytes at bare SPCE and ULP/SPCE electrodes. In all systems, the anodic peak current increased progressively with scan rate. No corresponding cathodic peak was detected on the reverse sweep over the investigated scan-rate range, supporting the irreversible character of the oxidation processes.

Linear relationships between peak current and the square root of the scan rate (Ip vs. *v*^1^ᐟ^2^) were obtained for both heterocyclic compounds, indicating predominantly diffusion-controlled oxidation processes [76,77]. For the ULP/SPCE electrode, the corresponding regression equations were Ip = 21.39*v*^1^ᐟ^2^ − 0.895 (R^2^ = 0.9856) for 2AO and Ip = 23.13*v*^1^ᐟ^2^ − 0.249 (R^2^ = 0.9889) for 2AT. In comparison, the bare SPCE electrode exhibited lower slopes for both analytes, confirming the improved interfacial electrochemical response promoted by the ultrasonically synthesized LaFeO_3_ material.

Additional kinetic information was obtained from the dependence of log(Ip) on log(*v*). The slopes obtained for all evaluated systems were close to 0.5, confirming diffusion-controlled irreversible oxidation behavior [76,77]. For ULP/SPCE, the linear relationships were log(Ip) = 0.4898log(*v*) − 0.125 (R^2^ = 0.9866) for 2AT and log(Ip) = 0.4425log(*v*) − 0.009 (R^2^ = 0.9875) for 2AO. Furthermore, the oxidation peak potentials shifted linearly with log(*v*), which is characteristic of irreversible electrode processes described by Laviron-type behavior [76,77,78]. According to Laviron’s model, the oxidation peak potential can be expressed as:

(3) $$E_{pa}=E^{0}+\left(\frac{2.30\;RT}{\alpha n\;F}\right)log\left(\frac{RT{\;k}^{0}}{\alpha n\;F}\right)+\left(\frac{2.30\;RT}{\alpha n\;F}\right)log(v)$$

where α is the charge-transfer coefficient, *k*^0^ is the standard heterogeneous rate constant, *v* is the scan rate, *E^0^* is the formal potential, *R* is the gas constant, *T* is the temperature, *F* is the Faraday constant, and n is the apparent number of electrons involved in the oxidation process. Application of Laviron’s model yielded αn values of 0.64 for 2AT and 0.63 for 2AO at ULP/SPCE, compared with 0.95 and 1.1 for 2AT and 2AO at bare SPCE, respectively. These results suggest modified charge-transfer behavior and possible stabilization of radical-cation intermediates at the LaFeO_3_-modified interface.

The linear dependence of oxidation potential on pH, together with the irreversible behavior identified from the scan-rate analyses, supports the participation of proton-coupled electron-transfer (PCET) processes during electrooxidation. Such behavior suggests that protonation equilibria of the amino-heterocyclic compounds significantly influence interfacial charge-transfer kinetics at the LaFeO_3_-modified electrode. Moreover, the Laviron-type behavior is consistent with an EC-type oxidation mechanism involving an initial electrochemical oxidation step followed by subsequent chemical transformations of radical-cation intermediates.

**Scheme 1:**
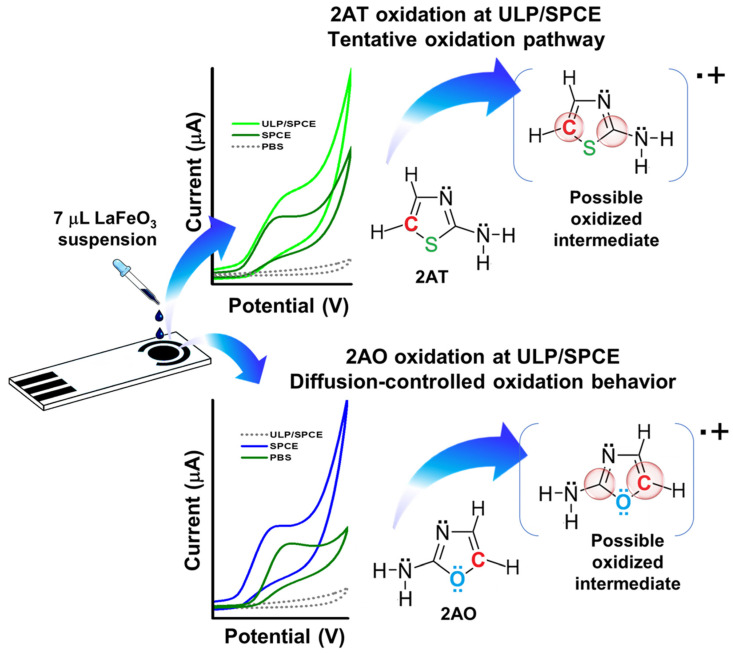
Tentative electrooxidation pathway of 2-aminothiazole (2AT) and 2-aminooxazole (2AO) at LaFeO_3_-modified SPCEs based on pH-dependent and scan-rate electrochemical analyses. The proposed pathway involves irreversible proton-coupled electron transfer followed by possible chemical transformations of the oxidized intermediates.

Based on the electrochemical behavior and scan-rate analyses, the oxidation of 2AT and 2AO at the LaFeO_3_-modified interface is proposed to proceed through an irreversible proton-coupled electron-transfer process involving the formation of radical-cation intermediates (Scheme 1). Subsequent chemical coupling reactions may occur after the initial electrochemical oxidation step, consistent with the EC-type behavior identified from the Laviron analyses. The differentiated oxidation responses observed for 2AT and 2AO suggest molecule-dependent stabilization of oxidized intermediates at the nanoperovskite interface.

Overall, the electrochemical analyses indicate that ULP/SPCE exhibited enhanced interfacial electron-transfer behavior toward the amino-heterocyclic compounds, as reflected by its larger electroactive area and lower charge-transfer resistance. The electrochemical results provide a tentative framework for interpreting the oxidation processes occurring at the LaFeO_3_-modified interfaces, while the exact identity and reaction pathways of the oxidized intermediates require further investigation.

## 3. Materials and Methods

### 3.1. Reagents and Chemicals

Lanthanum nitrate hexahydrate (La(NO_3_)_3_·6H_2_O, 99.9%, GFS Chemicals, Powell, OH, USA) and iron (III) nitrate nonahydrate (Fe(NO_3_)_3_·9H_2_O, 98%, Merck, Rahway, NJ, USA) were used as metal precursors for the synthesis of LaFeO_3_ nanoparticles. Commercial sugarcane molasses (Frutos y Semillas brand, Itagüí, Colombia) was employed as a biomass-derived fuel and organic carbon source. Potassium dihydrogen phosphate (KH_2_PO_4_, Rahway, NJ, USA) was used for the preparation of phosphate buffer solutions, and pH adjustment was carried out using 0.1 M KOH and diluted phosphoric acid (H_3_PO_4_, Merck, Rahway, NJ, USA). Potassium ferricyanide/potassium ferrocyanide ([Fe(CN)_6_]^3−^/^4−^) and KCl (Sigma-Aldrich, St. Louis, MO, USA) were employed as electrochemical redox probes for interfacial characterization experiments. 2-Aminothiazole (2AT) and 2-aminooxazole (2AO) (Sigma-Aldrich) were used as model heterocyclic compounds for electrochemical oxidation studies. All aqueous solutions were prepared using deionized water with a resistivity of 18.2 MΩ·cm.

### 3.2. Green Synthesis of LaFeO_3_ Nanoparticles

#### 3.2.1. Mechanically Stirred Combustion Synthesis

Lanthanum nitrate hexahydrate (2 mmol) and iron (III) nitrate nonahydrate (2 mmol) were separately dissolved in distilled water to obtain clear precursor solutions. The iron precursor solution was added dropwise to the lanthanum solution under continuous mechanical stirring and moderate heating to ensure homogeneous mixing.

Subsequently, an aqueous solution of sugarcane molasses, previously dissolved and homogenized at mild temperature, was added dropwise to the mixed-metal precursor. The resulting solution was heated until solvent evaporation and self-sustained combustion occurred, yielding a voluminous precursor powder. The as-combusted material was calcined in air at 900 °C for 5 h to obtain crystalline LaFeO_3_ nanoparticles. The resulting powder was gently ground and stored for further characterization. Samples obtained by this route are denoted as MLP.

#### 3.2.2. Ultrasonic-Assisted Combustion Synthesis

For ultrasonic-assisted synthesis, precursor solutions containing lanthanum and iron nitrates at the same molar ratio were subjected to ultrasonic irradiation prior to and during mixing in order to enhance precursor dispersion and homogenization. The iron precursor was added dropwise to the lanthanum solution under continuous ultrasonic agitation.

An aqueous solution of sugarcane molasses was then introduced under the same ultrasonic conditions. After ultrasonic treatment, the precursor mixture was transferred to a hot plate and heated to induce combustion, yielding a fine precursor powder. Calcination was carried out in air at 900 °C for 5 h to produce crystalline LaFeO_3_ nanoparticles. Samples obtained by this route are denoted as ULP.

### 3.3. Characterization Techniques

The phase composition and crystal structure of the synthesized materials were analyzed by X-ray diffraction (XRD) using a PANalytical Empyrean diffractometer (PANalytical, Almelo, The Netherlands) equipped with a scintillation detector and a parallel plate collimator operating with Cu Kα radiation (λ = 1.5406 Å). Diffraction data were further analyzed by Rietveld refinement in order to evaluate crystallographic parameters, phase composition, and microstructural characteristics of the synthesized LaFeO_3_ nanoperovskites.

Morphological features were examined by transmission electron microscopy (TEM) using a FEI Tecnai F20 Super Twin microscope (FEI Company, Hillsboro, OR, USA), and elemental composition was evaluated by energy-dispersive X-ray spectroscopy (EDS) using an EDAX Apollo X detector (EDAX Inc., Mahwah, NJ, USA). Inductively coupled plasma–optical emission spectrometry (ICP-OES) was additionally employed to evaluate the elemental composition and approximate stoichiometry of the synthesized materials.

Thermogravimetric analysis (TGA) and differential scanning calorimetry (DSC) were carried out to investigate the thermal stability and decomposition behavior of the combustion-derived materials. The measurements were performed using a Discovery SDT 650 thermal analyzer (TA Instruments, New Castle, DE, USA). Fourier-transform infrared (FTIR) spectroscopy was employed to identify characteristic metal–oxygen lattice vibrations and surface functional groups associated with residual biomass-derived surface species. FTIR spectra were recorded in the range 400–4100 cm^−1^ using a JASCO FT/IR-4100 spectrometer equipped with an ATR PRO450-S module (JASCO International Co., Ltd., Tokyo, Japan), with a spectral resolution of 4 cm^−1^ and 512 scans.

### 3.4. Fabrication of Modified SPCEs and Electrochemical Measurements

LaFeO_3_ nanoparticle dispersions were prepared by ultrasonic treatment of the synthesized powder in deionized water to obtain homogeneous suspensions. A 2 mg sample of LaFeO_3_ nanoparticle was dispersed in 1.5 mL of deionized water by ultrasonic agitation for 1 h. A Bioanalytical Systems, Inc., BAS Inc. (West Lafayette, IN, USA) single-compartment cell was employed. Screen-printed carbon electrodes (SPCEs, ItalSens Carbon SPE, PalmSens BV, Houten, The Netherlands) with a three-electrode configuration (working, counter, and reference electrodes) were used. The working and counter electrodes were composed of carbon ink, while the reference was a silver-based pseudo-reference [79,80]. For the electrochemical comparison of 2AT and 2AO at bare and ULP-modified SPCEs, a total of 12 fresh electrodes were used. Three independently prepared electrodes were evaluated for each of the four electrode/analyte conditions: bare SPCE + 2AO, ULP/SPCE + 2AO, bare SPCE + 2AT, and ULP/SPCE + 2AT (n = 3 per condition). The results are expressed as mean ± standard deviation.

Prior to modification, the electrodes were electrochemically activated via 10 cyclic voltammetry (CV) scans in 0.1 M phosphate buffer (PBS) from –0.1 to +1.2 V vs. Ag/AgCl to improve electron transfer kinetics. Modified electrodes were fabricated by drop-casting 7 μL of the LaFeO_3_ suspension (corresponding to approximately 9.3 μg of LaFeO_3_ per electrode) onto the working electrode surface and allowing the modified interfaces to dry at room temperature [73,80]. Direct AFM or SEM imaging of the deposited LaFeO_3_ layer was not performed; therefore, conclusions regarding electrode-surface coverage and local film morphology are limited to the controlled deposition protocol and the measured electrochemical interfacial response. Electrochemical measurements were performed using a Metrohm Autolab PGSTAT128N potentiostat/galvanostat (Metrohm Autolab B.V., Utrecht, The Netherlands) in a conventional single-compartment electrochemical cell. Cyclic voltammetry (CV), differential pulse voltammetry (DPV), and electrochemical impedance spectroscopy (EIS) were employed to evaluate the interfacial charge-transfer properties of bare and modified electrodes using the ferri/ferrocyanide redox couple as a standard electrochemical probe.

Additional electrochemical experiments involving amino-heterocyclic compounds (2AT and 2AO) were conducted under fixed experimental conditions using 0.5 mM solutions of each analyte in PBS. The potential was scanned from –0.1 to +1.2 V and then reversed to –0.1 V to investigate molecule-dependent interfacial oxidation behavior at the modified electrodes. The scan rate was varied from 0.025 to 0.40 V s^−1^ to investigate the electron-transfer kinetics, while the influence of pH (2.5–10.5) on the electrochemical response was systematically evaluated to elucidate the proton-coupled oxidation mechanism and interfacial electrochemical behavior of the modified electrodes.

These measurements were not intended to establish analytical sensing performance. Quantification was performed by differential pulse voltammetry (DPV) with a step potential of 5 mV, pulse amplitude of 100 mV, pulse width of 50 ms, and scan rate of 10 mV·s^−1^ [81]. Electrochemical impedance spectroscopy (EIS) was used to assess the charge-transfer resistance of the electrodes.

Each inter-electrode replicate was therefore performed using a fresh, independently prepared SPCE. Long-term storage stability and regeneration after analyte oxidation were not evaluated in the present study.

## 4. Conclusions

This study demonstrates that sugarcane-molasses-assisted combustion provides an effective and sustainable route for the synthesis of LaFeO_3_ nanoperovskites with tunable structural, microstructural, and interfacial electrochemical properties. The comparative evaluation between mechanically stirred and ultrasonic-assisted synthesis routes revealed that ultrasonic irradiation influences crystallization behavior, particle agglomeration, and electroactive area during biomass-assisted combustion synthesis.

Structural and microstructural analyses confirmed that the ultrasonically synthesized LaFeO_3_ material exhibited reduced crystallite dimensions, less compact agglomerated domains, and enhanced phase purity compared with the mechanically stirred counterpart. Thermal and vibrational analyses additionally confirmed the successful formation of thermally stable LaFeO_3_ nanoperovskites while revealing the presence of residual biomass-derived surface species associated with the combustion process.

Electrochemical characterization demonstrated that the ultrasonically synthesized material exhibited improved interfacial charge-transfer behavior, lower charge-transfer resistance, increased electroactive surface area, and enhanced electron-transfer kinetics at SPCE-based interfaces. Furthermore, the LaFeO_3_-modified electrodes displayed differentiated electrochemical oxidation responses toward nitrogen- and sulfur-containing heterocyclic compounds, confirming the sensitivity of the nanoperovskite interfaces to subtle molecular and interfacial interactions.

Overall, the present work establishes clear process–structure–property relationships linking sustainable synthesis strategies, nanostructural characteristics, and interfacial electrochemical behavior in LaFeO_3_-based perovskite systems. These findings provide comparative process–structure–property evidence that may guide future optimization of sustainable LaFeO_3_-based electrochemical interfaces.

## Figures and Tables

**Table 1:** Summary of LaFeO_3_ Nanoparticle Synthesis Conditions.

Sample	Molasse	La(NO_3_)_3_·6H_2_O	Technique	Fe(NO_3_)_3_·9H_2_O	Yield %	t/h	T/°C
	[g mL^−1^]	[g mL^−1^]		[g mL^−1^]			
MLP	0.20	0.17 (2.0 mmol)	Thermal Combustion	0.17 (2.0 mmol)	78	5 h	900
ULP	0.20	0.17 (2.0 mmol)	Ultrasonic-assisted combustion	0.17 (2.0 mmol)	86	5 h	900

**Table 2:** Structural parameters of LaFeO_3_ nanoparticles synthesized by ultrasonic-assisted (ULP) and mechanically stirred (MLP) combustion routes obtained from XRD and Rietveld refinement.

Sample	Parameter							
	a (Å)	b (Å)	c (Å)	Unit Cell Volume (Å^3^)	Rwp (%)	GOF	χ^2^	Average Crystallite Size (nm)
ULP ^a,b^	5.53749	7.83377	5.55313	240.892	15.34	5.92	23.81	70.62
MLP ^a,b^	5.53542	7.82957	5.55039	240.554	12.77	7.10	15.05	86.01

^a^ Crystal system: Orthorhombic, ^b^ Space group: Pnma.

**Table 3:** Peak positions, FWHM values, and crystallite sizes of ULP and MLP samples.

Sample ULP (Average D = 70.62 nm)			Sample MLP (Average D = 86.01 nm)		
Peak Location 2θ (°)	FWHM β (°)	Crystallite Size D (nm)	Peak Location 2θ (°)	FWHM β (°)	Crystallite Size D (nm)
22.69	0.11227	65.62	22.65	0.12483	87.84
32.31	0.13992	77.79	32.25	0.14222	85.37
39.84	0.16028	75.10	39.77	0.16465	88.58
46.34	0.20017	68.07	46.24	0.18508	82.24
57.62	0.24533	66.51	57.49	0.21351	86.00

**Table 4:** Comparison of representative synthesis routes and structural/electrochemical characteristics of LaFeO_3_ nanoparticles reported in the literature and the present work.

Synthesis Route	Fuel/Precursor	Calcination (°C/h)	Crystallite Size (nm)	Rietveld Refinement	Electrochemical Study	Reference
Sol–gel	Citric Acid	550/2 h	21.1–23.2	No	CA, EIS	[50]
Sol–gel	Citric Acid	800/6 h	15.8	No	No	[51]
Thermal decomposition	PVP	600/1 h	25–35	No	No	[52]
Sol–gel	Citric Acid	750/6 h	20–28	No	LSV	[53]
Green	Lemon	600/2 h	32.4	Yes	EIS	[54]
Hydrothermal	Citric Acid	600/5 h	39	No	CV, GCD	[55]
Co-precipitation	Ammonium carbonate	600/4 h	34.1	No	No	[56]
Solid-state reaction	-------	800/16–20 h	90–100	Yes	CV	[57]
Citrate sol–gel	Citric Acid	850/4 h	75	No	No	[58]
Ultrasound assisted combustion	Sugarcane molasses	900/5 h	70.6	Yes	CV, DPV, EIS	**This work**

**Table 5:** Peak current and potential values for 2AT and 2AO oxidation at SPCE and ULP/SPCE in PBS (pH 6.0).

Surfaces	Peak Current Ipa (µA)	Peak Potential Epa (V)
SPCE + 2AO	3.16 ± 0.29	0.755 ± 0.026
ULP/SPCE + 2AO	3.75 ± 0.34	0.686 ± 0.031
SPCE + 2AT	4.02 ± 0.32	0.663 ± 0.034
ULP/SPCE + 2AT	2.15 ± 0.27	0.725 ± 0.021

Values are expressed as mean ± standard deviation from three independently prepared electrodes (*n* = 3) per electrode/analyte condition. Both analytes have pKa values near 5.3–5.5 [73].

## Data Availability

Data will be made available on request.
